# The Late Positive Potentials Evoked by Cigarette-Related and Emotional Images Show no Gender Differences in Smokers

**DOI:** 10.1038/s41598-019-39954-0

**Published:** 2019-03-01

**Authors:** Elise M. Stevens, David Frank, Maurizio Codispoti, George Kypriotakis, Paul M. Cinciripini, Kimberly Claiborne, Menton M. Deweese, Jeffrey M. Engelmann, Charles E. Green, Maher Karam-Hage, Jennifer A. Minnix, Jennifer Ng, Jason D. Robinson, Rachel F. Tyndale, Damon J. Vidrine, Francesco Versace

**Affiliations:** 1 Stephenson Cancer Center, Oklahoma Tobacco Research Center, University of Oklahoma Health Sciences Center, Oklahoma City, OK USA; 2 MD Anderson Cancer Center, University of Texas, Houston, TX USA; 3 Dipartimento di Psicologia, Universita’ di Bologna, Bologna, Italy; 4 Department of Pediatrics, University of Texas, Houston, TX USA; 5 Centre for Addiction and Mental Health, University of Toronto, Toronto, Ontario Canada; 6 Medical College of Wisconsin, Milwaukee, WI USA; 7 Vanderbilt University, Department of Teaching and Learning, Nashville, Tennessee USA

## Abstract

When trying to quit, women are less likely than men to achieve long-term smoking abstinence. Identifying the neuropsychological mechanisms underlying women’s higher relapse vulnerability will help clinicians to develop effective tailored smoking cessation interventions. Here we used event-related potentials (ERPs), a direct measure of brain activity, to evaluate the extent to which neurophysiological responses to cigarette-related and other emotional stimuli differ between female and male smokers. Both women and men showed similar patterns of brain reactivity across all picture categories; pleasant and unpleasant images prompted larger Late Positive Potentials (LPPs, a robust measure of motivational relevance) than neutral images in both groups, and cigarette-related images prompted lower LPPs than high arousing emotional images in both groups. Unlike previous studies, there were no differences between male and female smokers with regard to LPP responses to cigarette-related images. This suggests that the LPP may not be ideally suited to discriminate neurophysiological gender differences or that there are simply no gender differences in the neurophysiological responses to cigarette-related stimuli. We collected ERPs from 222 non-nicotine-deprived smokers (101 women) while they watched a slideshow that included high and low emotionally arousing pleasant and unpleasant pictures, cigarette-related, and neutral pictures. We used the mean amplitude of the LPP to assess the affective significance that participants attributed to these pictures.

## Introduction

While smoking is on the decline in the United States, it remains the leading cause of preventable deaths^1^. In general, smokers find quitting very difficult: fewer than 8% of smoking cessation attempts (assisted or not) result in abstinence periods of more than six months^2^. Furthermore, women have a harder time achieving long-term abstinence than men^3^. Understanding the neuropsychological mechanisms underlying women’s higher vulnerability to relapse will help clinicians to better tailor, and thus improve, relapse prevention interventions^4,5^.

Results from previous studies suggest that cigarette-related cues, unpleasant, and stressful stimuli evoke stronger cravings in female smokers than male smokers^6–10^. However, these findings were mainly obtained through self-reports while smokers were exposed to a limited array of stimuli (e.g., only cigarette-related and neutral stimuli). While self-report offers an important window into the individual’s subjective experience, it does not provide information about the neuropsychological underpinnings of these experiences, therefore depriving researchers of information that can be used to develop new relapse prevention treatments^11^. Furthermore, if just cigarette-related and neutral stimuli are included in cue-reactivity experiments, researchers cannot draw strong conclusion about the meaning or the strength of the responses evoked by cigarette-related stimuli^11,12^. By including other motivationally relevant stimuli, varying both valence and arousal, researchers can determine the strength of the responses evoked by cigarette-related cues in relation to other non-drug-related motivationally relevant stimuli and better test the hypothesis that cigarette-related cues “hijack human brain motivational systems and promote drug seeking over alternative behaviors”^11^.

Here, we used event-related potentials (ERPs), a non-invasive measure of neural activity^13,14^, to examine the extent to which male and female smokers differ in their neuroaffective responses to cigarette-related, emotional, and neutral cues. Specifically, to assess the affective significance of the stimuli, we used the amplitude of the Late Positive Potential (LPP), one of the most robust and replicable neurophysiological indices of motivational relevance. The LPP is an ERP component that peaks between 400 and 800 milliseconds (ms) after stimulus onset over central and parietal electrode sites^15–17^. Both pleasant and unpleasant images increase the amplitude of the LPP above the level measured for neutral images^15,17–21^. The LPP modulation has high temporal stability^22^, is particularly pronounced for highly arousing pictures (e.g., erotica or mutilations)^21,23^, and is resistant to manipulations affecting perceptual composition^24–26^, exposure time^27^, and stimulus repetition^28,29^. These results indicate that the LPP amplitude mainly reflects the extent to which a visual stimulus engages the motivational brain circuits^30–32^.

In line with the idea that drug-related cues can become highly significant for chronic users^33^, several studies showed that, in smokers, cigarette-related images prompt larger LPPs than neutral images^34–37^, even though, on average, the LPP evoked by cigarette-related cues is smaller than the LPP evoked by highly arousing emotional images^38,39^ (e.g., erotica and mutilations). Hence, by including high and low arousing pleasant and unpleasant stimuli, in addition to cigarette-related and neutral stimuli, the current study aimed at providing a more nuanced assessment of the neuropsychological responses that might be responsible for the higher relapse vulnerability that women experience.

Furthermore, by including a wide array of pleasant and unpleasant stimuli, this study also offers the opportunity to investigate the extent to which genders differ in the way they process emotional stimuli, a controversial issue in affective neuroscience. In Western culture, a persisting stereotype is that women are more emotional than men, and particularly more reactive to unpleasant events, while men are thought to be more reactive to pleasant stimuli^40–42^. Some observations support this stereotype: Women report higher scores on scales related to negative emotional experiences and have increased risk of suffering from depression and anxiety disorders^43^. Previous studies^44,45^ also found that compared to men, women rated unpleasant stimuli as more arousing and unpleasant, while men rated erotic materials as more arousing and pleasant. However, given that social desirability/appropriateness and sociocultural factors can drive subjective ratings, researchers tried to investigate gender differences in emotional reactivity using ERPs. That is because neurophysiological responses reflect motivational engagement more directly and are less likely to be driven by sociocultural factors. However, these ERP studies mainly recruited small samples of college students and yielded inconsistent findings regarding LPP affective modulation^46–51^.

Thus, in the present study we aimed to examine gender differences in LPP affective modulation assessing a large sample of middle-aged adults while they were looking at emotional, cigarette-related, and neutral pictures.

## Results

### Participant Characteristics

There were no significant differences between male and female smokers on nicotine dependence (measured by the FTCD), positive affect, negative affect, or depressive symptomatology (measured by the CES-D). However, female smokers (*M* = 48.58) were older than male smokers (*M* = 45.42; *p* < 0.05) and reported (*M* = 42.65) greater smoking urges (measured by the Brief Questionnaire of Smoking Urges- QSU) than male smokers (*M* = 35.36; *p* < 0.05; Table 1).

**Table 1:** Participant Demographics.

Measure	Women (n = 101)		Men (n = 121)		All (n = 222)	
	Mean	(SD)	Mean	(SD)	Mean	(SD)
Age	48.5*	(9.9)	45.4*	(10.5)	46.9	(10.3)
FTCD	4.7	(1.9)	4.9	(2.0)	4.8	(2.0)
CO (ppm)	16.27	(9.24)	17.91	(9.17)	16.94	(9.1)
Time since last cigarette	2:30	(3:59)	2:20	(3:34)	2:25	(3:42)
QSU-B	42.7*	(27.1)	35.4*	(24.9)	38.7	(26.1)
PANAS Positive Affect	36.1	(7.5)	32.2	(8.1)	35.1	(7.9)
PANAS Negative Affect	16.4	(5.6)	17.8	(7.0)	17.2	(6.4)
CES-D	10.7	(7.9)	11.4	(8.2)	11.1	(8.1)

Note: *p < 0.05. FTCD = Fagerström Test for Cigarette Dependence, CO = expired carbon monoxide, ppm = parts per million, Time since last cigarette = hours: minutes, QSU-B = Brief Questionnaire of Smoking Urges, PANAS = Positive And Negative Affective Schedule, CES-D = The Center for Epidemiologic Studies Depression Scale.

### Late Positive Potential Main Analysis

**Figure 1:**
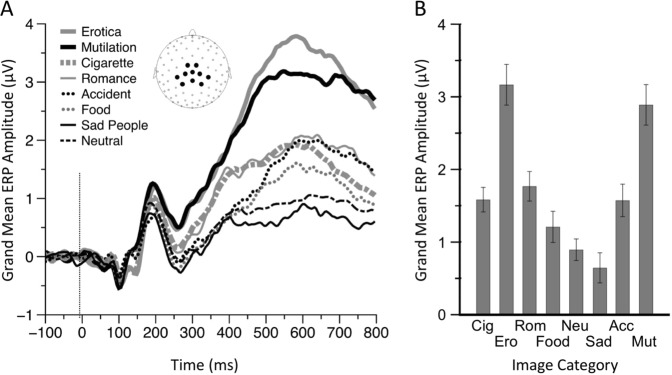
Affectively significant images (including cigarette-related images) prompt larger Late Positive Potentials (LPPs) than neutral images. Cigarette-related images prompt lower LPPs than high arousing emotional images. Panel A depicts ERP waveforms for each picture category (averaged across both women and men). The box highlights the time region of interest (ROI) used to compute the LPP across the electrodes highlighted in the inset. Panel B shows mean LPP from 400 to 800 ms post stimulus onset for each picture category. Note: Cig = Cigarettes, Ero = Erotic, Rom = Romantic, Neu = Neutral, Sad = Sad People, Acc = Accidents, Mut = Mutilations. Error bars denote 0.95 confidence intervals.

**Figure 2:**
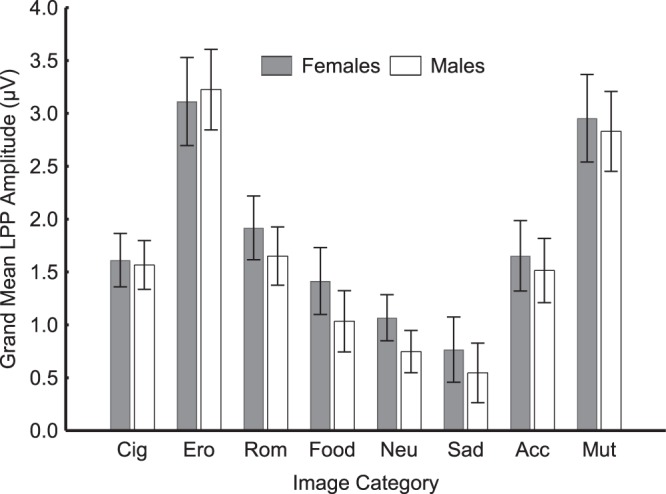
Both women (grey bars) and men (white bars) show comparable patterns of reactivity. In both genders, the LPP amplitude increases as a function of the affective significance of the visual stimuli, regardless of their hedonic valence. On average, cigarette-related cues evoke LPP responses more similar to those evoked by low-arousing emotional stimuli than high-arousing ones. Note: Cig = Cigarettes, Ero = Erotic, Rom = Romantic, Neu = Neutral, Sad = Sad People, Acc = Accidents, Mut = Mutilations. Error bars denote 0.95 confidence intervals.

Figure 1(A) shows the ERP waveforms, averaged across women and men, for each picture category, and the time window used to calculate the amplitude of the LPP. The main effect of image category was significant [*F*(7, 1540) = 99.88, *p* < 0.0001; see Fig. 1, panel B]. Bonferroni-corrected pairwise comparisons confirmed that the LPP evoked by neutral stimuli was significantly (*p* < 0.0001) smaller than the LPP evoked by every image category except “food” and “sad people.” Erotic scenes and mutilation scenes evoked LPPs were significantly larger than LPPs evoked by every other picture category (*p* < 0.0001), but were not significantly different from each other (p = 0.62). Cigarette-related stimuli evoked LPPs were not significantly different from those evoked by low arousing stimuli (i.e., romantic, food, and violence), even though the difference between cigarette-related and food stimuli was trending towards significance, with cigarette-related evoking greater LPPs (*p* = 0.07). As the non-significant interaction gender by stimuli category indicates (*F*(7, 1540) = 0.80, *p* = 0.59), women and men exhibited similar brain reactivity patterns (Fig. 2). If no correction for multiple comparisons was applied, the only difference between genders was the LPP response to neutral stimuli (*p* = 0.03). To control for this difference, we run a secondary, exploratory analysis of covariance using the LPP amplitude evoked by neutral images as covariate, gender as a between-subjects factor, image category (cigarette-related, erotic, romantic, food, sad people, accidents, and mutilations) as a within-subjects factor. The results of this analysis replicated those presented above: women and men exhibited similar brain reactivity patterns across all stimulus categories. Moreover, we computed the Bayes factor of the model omitting the interaction effect of gender with stimuli category (in the numerator) against the model including the interaction term (in the denominator). Consistent with the classical ANOVA results, the analysis yielded a Bayes factor of 607.4 (error = 2.4%), indicating very strong evidence^52^ against the model with the gender by stimulus category interaction.

## Discussion

In this study, we used the amplitude of the LPP, a valid and robust neurophysiological index of affective reactivity, to examine the extent to which gender differences influence responses to cigarette-related and non-cigarette-related emotional stimuli. We replicated previous studies showing that visual stimuli increase the LPP amplitude as a function of their affective significance, regardless of their hedonic valence^13,15,19–21,30–32^. We also replicated our previous findings^38,39^ showing that, on average, cigarette-related cues evoke LPP responses that are more similar to those evoked by low-arousing emotional stimuli than to those evoked by high-arousing ones. Finally, we demonstrated that, even if women reported higher levels of tonic cravings than men did, both genders had very similar LPP responses to cigarette-related and to non-cigarette-related emotional stimuli.

Our results are consistent with those from previous studies that failed to detect differences between genders in reactivity to cigarette-related cues when using measures of peripheral nervous system activation^9^ and self-reports^10^. Specifically, Saladin and colleagues^9^ detected no heart rate or skin conductance differences between female and male smokers in response to smoking cues. Likewise, Colamussi and colleagues (2007)^10^ found that male and female smokers did not report significantly different levels of craving after reading about a smoking situation (e.g., lighting up after a meal). Although, these studies did not specifically focus on measures of central nervous system activity.

The evidence for gender differences in reactivity to cigarette-related stimuli using direct or indirect measures of central nervous system activity is limited; two studies have found that men exhibit larger fMRI responses to cigarette-related cues compared to women^53,54^. Our data indicate that, even though women reported higher levels of tonic cravings than men did, they did not have larger neurophysiological responses to cigarette-related cues, suggesting that the higher relapse rates observed in women might not be due to higher neurophysiological reactivity to cues. Furthermore, the lack of gender differences in reactivity to both high and low arousing non-cigarette-related pleasant and unpleasant stimuli also suggests that, unlike previously hypothesized, higher reactivity to unpleasant stimuli might not be responsible for the higher relapse rates that women have when trying to quit smoking.

Previous studies about gender differences in the processing of pleasant and unpleasant stimuli have yielded inconsistent findings as well^46–51^. These inconsistencies may have to do with differences in the emotional contents of the stimuli used across studies, and with the inclusion of small samples of college students (on average, previous studies included approximately 30 individuals per group), and therefore low statistical power. Button and colleagues^55^ recently showed that statistically significant results from low powered studies are not likely to actually reflect a true effect. Our study addressed these weaknesses by comparing two relatively large samples of women (*n* = 101) and men (*n* = 121), and by measuring responses to a wide array of standardized visual stimuli belonging to both pleasant and unpleasant categories with different levels of emotional arousal. In the present study, pleasant and unpleasant stimuli elicited similar LPP responses in women and men. Given the characteristics of the LPP (i.e., its high temporal stability^22^, it resistance to manipulations affecting perceptual composition^24,25^, exposure time^27^, and stimulus repetition^28,29^), this result suggests no gender differences in attentional and motivational processing of emotional visual stimuli. Our findings are in line with two fMRI meta-analyses indicating that men and women have very similar patterns of activation in the presence of both pleasant and unpleasant stimuli: The only trends highlighted by these meta-analyses were a somewhat larger response of women (compared to men) to pleasant and unpleasant stimuli in inferior temporal (IT) cortex, and, only for unpleasant stimuli, in the amygdala^56,57^. Even these differences might be the result of publication and other reporting biases in the literature, as a recent study focusing on the fMRI literature on functional sex differences in human brain cognitive functions highlighted^58^.

On the other hand, our findings should not necessarily lead to the conclusion that men and women do not differ in other processes implicated in emotional reactivity. In fact, using self-report measures, Ferrari and colleagues showed that women tend to rate erotic images as less emotionally arousing than men^40^; a pattern that has been observed also using measures of central nervous system activation, albeit in small samples of participants^59,60^. Thus, while there might be small gender differences in the level of activation that motivationally charged stimuli prompt in circumscribed brain regions, our findings indicate that men and women would appear to be more similar than they are different.

One limitation of our study includes the fact that cigarettes were not actually available during the experiment, a condition that might blunt reactivity to cigarette-related stimuli^61,62^. Another potential limitation of our study is that we collected all the data when participants were not nicotine deprived: on average, the session took place less than three hours since participants smoked their last cigarette. It is possible that nicotine deprivation may affect men and women differently with regard to neurophysiological reactivity to cues, however the results of a previous study, where we recorded ERPs in nicotine deprived female and male smokers^63^, suggest that when nicotine deprived, male and female smokers react similarly to emotional and cigarette-related cues. Our study also might be limited by the fact that we did not control for menstrual cycle phase for female participants, as that may have the potential to affect brain reactivity^64^. Finally, it is important to note that we did not measure self-reported cue-induced craving on a trial-by trial basis during the experiment. As the self-reports of tonic craving suggest, it is possible that the craving experience induced by cues might differ between women and men even if neurophysiological responses are similar^65^.

Limitations notwithstanding, this study is an important step in evaluating gender differences in reactivity to cigarette-related and other emotional cues. Our analyses align with the National Institutes of Health (NIH) expectation that scientists should account for the possible role of sex as a biological variable in human studies (NOT-OD-15-102)^66^. Hence, as the results reported by David and colleagues^58^ highlight, it is imperative that null findings are reported and published to avoid biasing the literature^67^. In summary, even though phasic electrophysiological responses to emotional and cigarette-related cues do not distinguish male and female smokers, as self-reported tonic craving indicates, other factors may differentiate the genders. Future work should identify these factors in an attempt to reduce the higher vulnerability to relapse that women experience when they try to quit.

## Material and Methods

### Participants

We recruited 222 smokers (45% women) from the general community of Houston, Texas metropolitan area using television, newspaper, and radio advertising, in addition to distributing flyers. Inclusion criteria were: aged between 18 and 65 years old, fluent in English, smoking more than 10 cigarettes per day for at least the last six months, and having a baseline expired carbon monoxide (CO) level greater than 10 parts per million (ppm). Exclusion criteria included having a neurological, psychiatric, or substance abuse disorder (not including smoking), currently using psychotropic medication, actively trying to quit smoking, using non-cigarette tobacco products, history of substance abuse, or medical contraindications (pregnancy, history of cancer, kidney or liver disease or transplant, clinically significant cardiac dysrhythmias, stroke, angina, heart attack, or uncontrolled hypertension).

### Laboratory Session

Participants completed a telephone pre-screening interview aimed at assessing inclusion and exclusion criteria, then, if eligible, scheduled an in-person laboratory session. At the session, a trained staff member explained the study to the participant and, after obtaining informed consent, administered a computer-assisted self-report test battery aimed at assessing mood measured by the Positive and Negative Affect Schedule (PANAS)^68^ and Center for Epidemiologic Studies Depression Scale (CES-D)^69^. Additional information including demographics, such as age, gender, and smoking-related variables, such as the brief version of the Questionnaire of Smoking Urges^70^ and the Fagerström Test for Cigarette Dependence^71^ (FTCD; See Table 1), were also collected. Then, researchers placed sensors for the electroencephalogram (EEG) on the head of the participant and the participant watched a slideshow that included emotional, neutral, and cigarette-related stimuli. At the end of the slideshow, the participant was debriefed and received a $60 gift card for participating. MD Anderson Cancer Center Institutional Review Board approved all procedures and all methods, and those were performed in accordance with the relevant guidelines and regulations.

### Materials

During the EEG session, participants viewed a slideshow comprising eight categories of images: cigarette-related, erotic, romantic, food, neutral, accidents, sad people, and mutilations. These stimuli were selected from the International Affective Picture System (IAPS)^72^, from collections of smoking related images^73,74^, and from other collections used in previous studies^24–26,39^. During the slideshow, the stimuli appeared in pseudo-random order, with no more than two consecutive pictures from the same category. Each picture appeared on the screen for four seconds. A black screen with a white fixation cross at the center of the screen was presented after each picture for three to five seconds. Three, ten-minute blocks made up the slideshow, with the whole session lasting approximately 30 minutes. The slideshow ran on a 42” plasma television screen placed at a distance of 1.5 meters from the participant. The pictures subtended approximately a 24-degree visual angle. The slideshow was controlled by E-Prime software (version 1.4; Psychology Software Tools, PST Inc., Pittsburgh, PA) installed on a Pentium 4 computer.

### Data Reduction

We collected ERPs using a 129-channel Geodesic Sensor System (Geodesic EEG System 250; Electrical Geodesics, Inc., Eugene, OR). The data reduction procedures were the same as in our previous studies^36,37,39^. Data were filtered offline using a 30-Hz low pass filter and then inspected visually for the presence of artifacts. Channels contaminated by artifacts for more than 50% of the recording were interpolated using spherical splines. Eye blinks were corrected with BESA (version 5.1.8.10; MEGIS Software GmbH, Grӓfelfing, Germany) using a spatial filtering method. Data were then transformed to the average reference, segmented in 900 ms epochs starting 100 ms before picture onset and baseline corrected using the first 100 ms. Artifacts were identified by means of an automatic algorithm that used gradient (25 µV/ms maximal allowed voltage step), difference (100 µV maximal allowed difference within a segment), and amplitude (±100 µV absolute voltage allowed within a segment) criteria, and channels contaminated by artifacts in more than 40% of the segments were interpolated using six neighboring channels. We discarded segments with more than 10% of channels containing artifacts. Segments were then averaged separately for each category and the LPP was computed by averaging the voltage between 400 and 800 ms post picture onset across the following 10 centroparietal sensors EGI electrodes: 7, 31, 37, 54, 55, 79, 80, 87, 106, 129 (see inset of Fig. 1 for topographic location). The time and spatial regions of interest for the LPP were a-priori selected on the basis of previous studies^37^.

### Statistical analyses

To analyze self-reported participant characteristics, we ran a series of independent samples t-tests in which gender was the independent variable and QSU, FTCD, PANAS positive affect, PANAS negative affect, CES-D, and age were dependent variables, respectively.

To examine the LPP responses, we used Analysis of Variance (ANOVA) with gender as a between-subjects factor and image category (cigarette-related, erotic, romantic, food, neutral, sad people, accidents, and mutilations) as a within-subjects factor. To test for the presence of within and between groups differences, we used Bonferroni corrected pairwise comparisons to control for Type I errors.

## Data Availability

Materials, data, and associated protocols will be available to readers on request without undue qualifications.
